# Early diabetes screening via red blood cell mechanics using microfluidic chip integration^☆^^☆☆^

**DOI:** 10.1016/j.mbm.2025.100136

**Published:** 2025-05-29

**Authors:** Yibo Feng, Bingchen Che, Yonggang Liu, Cangmin Zhang, Jiameng Niu, Jiangcun Yang, Guangyin Jing, Dan Sun, Xiaobo Gong, Ce Zhang

**Affiliations:** a State Key Laboratory of Photon-Technology in Western China Energy, Institute of Photonics and Photon-Technology, Northwest University, No. 1, Xuefu Avenue, Xi'an, 710127, Shaanxi, China; b Laboratory of Stem Cell and Tissue Engineering, Chongqing Medical University, 400016, Chongqing, China; c WONGENY Precision Technology Co., Ltd., No. 169 Liaobang Road, Suzhou, 215217, Jiangsu, China; d Department of Transfusion Medicine, Shaanxi Provincial People's Hospital, Xi'an, 710068, Shaanxi, China; e School of Physics, Northwest University, No. 1 Xuefu Avenue, Xi'an, 710127, Shaanxi, China; f School of Ocean and Civil Engineering, Shanghai Jiao Tong University, Shanghai, 200240, China

**Keywords:** Reversal shear flow, Mechanical stimuli, Diabetes, Early screening

## Abstract

Early diagnosis of diabetes is crucial, as diabetes, particularly type 2, can eventually lead to irreversible changes and complications. Conventional techniques, such as the Fasting Plasma Glucose (FPG) Test and Hemoglobin A1c (HbA1c) Test, measure blood glucose levels, which fluctuate over time and are insensitive to early stages. In this study, we focus on measuring the mechanical properties of red blood cells, as their irreversible changes can indicate early pathological impacts of diabetes. We developed a microfluidic chip with a symmetrical hyperbolic structure. By periodically altering the state of the valve membrane, we generate a reciprocating shear flow field that repeatedly acts on groups of RBCs. We then quantify the morphological parameters of the RBCs, establishing a correlation between the reciprocating shear flow field and the morphological changes of the cells. Using the developed microfluidic chip, we investigated the resistance of blood cells from 20 healthy volunteers to mechanical stimuli. The results indicated a significant correlation between the deformability of red blood cells and age, while no such correlation was found among individuals of the same gender. This study highlights the potential of utilizing the mechanical properties of red blood cells as an early diagnostic tool for diabetes. Furthermore, given the ease of integration of microfluidic chips, they present a promising high-throughput diagnostic solution for large-scale clinical screening.

## Graphical abstract

**Figure:**
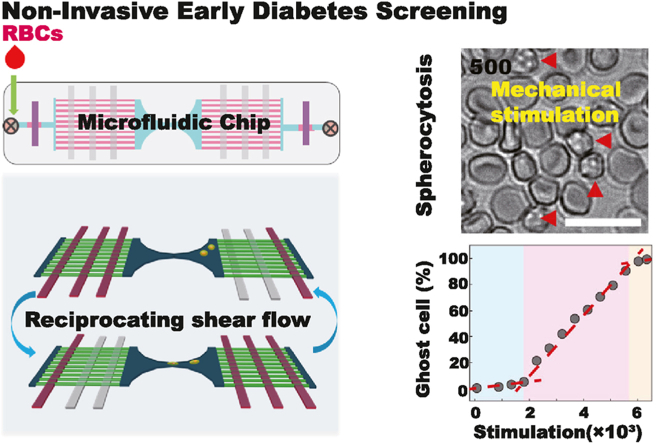

## 1 Introduction

Diabetes is a rapidly increasing chronic metabolic disease worldwide. According to data provided by the International Diabetes Federation, there are over 415 million diabetes patients globally, and this number is expected to continue rising in the coming years. ^1^^,^^2^ Early screening for diabetes has become crucial in order to detect and control the disease as early as possible. Currently, common methods for early screening of diabetes rely mainly on blood biochemistry markers and physical examinations. The primary techniques include the Fasting Plasma Glucose (FPG) Test and the Hemoglobin A1c (HbA1c) Test. ^3^ The FPG test measures blood glucose levels after an overnight fast, providing a snapshot of glucose metabolism. ^4^^,^^5^ The HbA1c test, on the other hand, measures the percentage of glycated hemoglobin in the blood, reflecting average blood glucose levels over the past two to three months. ^6^ Even though both methods are widely used, their diagnostic accuracy for early-stage diabetes is limited. The FPG test can be influenced by short-term factors such as recent food intake or stress, leading to fluctuations in glucose levels. 7, 8, 9 The HbA1c test, though more stable over time, may not detect short-term changes in blood glucose and can be affected by hemoglobin variants or conditions affecting RBCs. ^10^^,^^11^ Therefore, finding a convenient, and quick method for early screening of diabetes has become a key research focus.

Studies have shown that abnormal expression of RBC proteins in diabetic patients results in a reduced ability of RBCs to deform and may lead to morphological abnormalities ^12^^,^^13^ Investigating RBC shape changes in the context of blood flow in vivo, and analyzing their morphological alterations under shear stress, could serve as a novel biomarker for early changes associated with diabetes. Agrawal et al. ^14^ assessed the morphological parameters of RBCs using digital microscopy, while Chang et al. ^15^ evaluated RBC deformability through the deformability index. Both studies highlighted the significant role of altered RBC morphology in the onset and progression of diabetes. Furthermore, numerous studies have examined the effects of mechanical compression or stretching on RBC morphology. ^16^ Stenotic channels have been used in previous studies to investigate RBC deformability. For example, Wei et al. ^17^ combined hydrodynamic models and machine learning to study RBC mechanical properties under stress in micro circular tubes. Wang et al. ^18^ classified RBC dynamic behavior in microchannels, while McWhirter et al. ^19^ explored RBC shape transitions in cylindrical microcapillaries. However, these models fail to fully replicate the in vivo blood flow conditions, where there is no direct mechanical contact with the cells, and they are unable to accurately simulate the dynamic, complex environment that RBCs encounter in the circulatory system.

Reversal shear flow is a phenomenon in which fluid motion is induced by periodically applying forward and reverse shear stress. ^20^^,^^21^ In this study, we developed a microfluidic chip with a symmetrical hyperbolic structure integrated with Quake's valve membrane, 22, 23, 24 introducing a dynamic microfluidic environment that replicates the complex, oscillatory shear stresses and pressure fluctuations RBCs experience in vivo. By adjusting the deformation of the valve membrane, we can generate a stable and controllable flow field. The valve membrane's state can be periodically changed using a customized MATLAB program, creating a reciprocating shear flow field that continuously alters the shape of RBCs. By observing changes in the morphological parameters of RBCs in this reciprocating flow field, we establish correlations between RBC morphological changes and the shear flow conditions. This approach offers a new method for early screening of diabetes, aiming to improve diagnostic accuracy and providing a novel perspective for early diagnosis in the field of diabetes. Meanwhile, the integration of multiple hyperbolic structures in the chip significantly improves the detection throughput, providing possibilities for large-scale screening of diabetes. ^16^^,^^25^

## 2 Material and methods

### 2.1 Design and production of the chips

In this study, AutoCAD is utilized for designing chip templates, which are fabricated using SU-8 3025 and AZ-50X photoresists through standard UV lithography. AZ-50X is employed to create liquid channels with curved profiles to achieve rapid and effective closure of microvalves within the chip. This structure, in conjunction with rectangular fluidic channels etched from SU-8 3025, collectively forms the sample experimental layer. The control pressure layer, responsible for applying pressure to close the valves, is manufactured using SU-8 3025. The chip is fabricated using soft lithography techniques based on the prepared templates. During fabrication, 45g of PDMS (mixed at a ratio of 10:1 with curing agent) is poured onto the experimental layer mold, degassed in a vacuum chamber for 1 ​h, and baked at 80 ​°C for 2 ​h. Similarly, PDMS mixed at the same ratio is poured onto the control pressure layer, spin-coated at 2000 ​rpm for uniform distribution, baked at 80 ​°C for 10 ​min, followed by plasma treatment of both layers together for bonding. The bonded chip is subsequently baked for 8 ​h before use.

### 2.2 Chip operation

After preparing the fabricated chip through pressure testing, it undergoes sterilization. During operation, liquid lines controlled by micro-pneumatic solenoid valves are connected to various interfaces of the chip's control layer. MATLAB control programs are used to manage these solenoid valves. Target samples are introduced into the chip's experimental layer inlet using a pipette. Subsequently, the outermost two microvalves of the experimental chamber are closed to create a sealed environment within the chip.

### 2.3 Numerical simulation

We conduct numerical simulations using COMSOL Multiphysics 5.3. Based on the actual dimensions of the chip, 2D schematics are generated using CAD software and imported. The inlet flow velocity is set to 0.25 ​mm/s, and the middle channel is set to 5 ​mm. This simulation utilizes laminar flow physics to study transient results, using probes to obtain velocity data at various points. Under ideal conditions (i.e., low Reynolds numbers), Stokes' law can be applied to estimate the forces acting on RBCs based on fluid dynamics and the size of the RBCs. 26, 27, 28 Using these principles, we utilized the simulated fluid velocity distribution to assess the forces acting on the RBCs.

### 2.4 Image acquisition and data analysis

Single-donor RBCs were used for the experiment. 20 volunteers were recruited, and informed consent was obtained from all donors. The study was approved by the Ethics Committee of Shaanxi Provincial People's Hospital [(2020) R005]. Blood sample were collected from each individual, with a volume of 4–5 mL per donation. A 5 μL blood sample was diluted to 50 mL using 0.9% saline, and then 100 μL of the diluted blood sample was loaded into the microfluidic chip via pipetting. Images are acquired using a Nikon Ti2-Eclipse microscope, equipped with an automated stage and a digital CMOS camera (ORCA-Flash 4.0, Hamamatsu, Japan). Image capture is controlled by Nikon's microscope software (NIS Elements). Custom MATLAB programs are used for image analysis.

## 3 Results and discussion

### 3.1 Pressure gradient in the funnel-shaped microfluidic channel

**Fig. 1:**
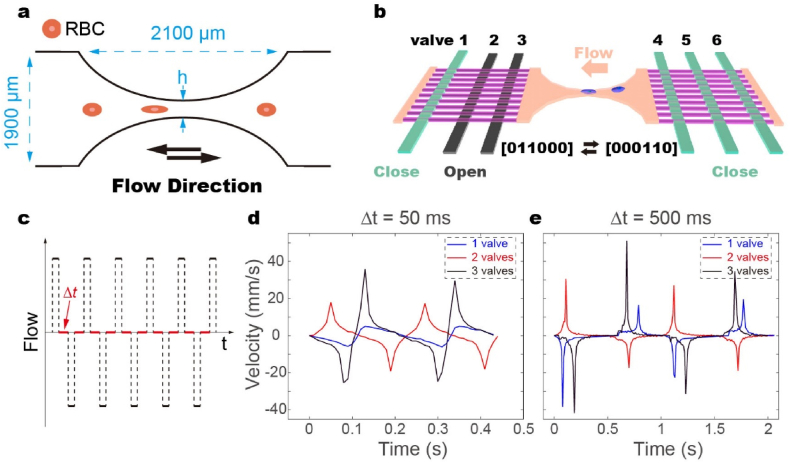
Reciprocating flow field generated by cycling operation of PDMS membrane valves. a. Hyperbolic symmetrical microchannels are 1900 ​μm wide and 2100 ​μm long, with a variety of sizes at the narrowest h (e.g., 5, 10, 15, 20, …, 100 ​μm). b. Schematic shows that the reciprocating flow field is generated by repeated operation PDMS membrane valves, i.e., the open/close of valve 2, 3, 4 and 5. c. The time-span required for valve to be fully closed is ∼50 ​ms. With defined 50 ​ms interval between valve operation, the cycling time of reciprocating flow is 200 ​ms. d,e. Variations in flow velocity at the center of constriction during repeated valve operation. It is clear that the flow rate is proportional to the number of involved valves.

RBCs experience shear stress and pressure variations induced by blood flow within blood vessels, which influence their deformation and behavior in the absence of direct mechanical contact. 29, 30, 31 To better simulate the in vivo environment, we employed a funnel-shaped microfluidic channel, where the fluidic shear stress and pressure gradients are well-defined by the channel geometry. The height and width of the funnel are 2.1 ​mm and 1.9 ​mm, respectively (Fig. 1a). Averaged dimension of the constriction (i.e., h) ranges from 5 ​μm to 100 ​μm. Numerical simulations using COMSOL were conducted to analyze the velocity distribution of the fluid across different regions of the channel. These simulations, which modeled pure fluid flow without the inclusion of RBCs, showed that with an unchanged input flow velocity of 0.250 ​mm/s, the pressure gradient reaches its maximum at the center of the constriction, where the velocity is 141.5 ​mm/s. This high velocity induces significant stretching of the cells (Fig. S2), allowing us to assess the mechanical forces on RBCs based on the simulated fluid velocity distribution. This approach provides a clearer understanding of the fluid dynamics and forces acting on RBCs.

To avoid inconsistency caused by external disturbance, the flow field is generated in the microfluidic chip by manipulating the integrated Quake's membrane valve. By controlling the opening and closing of 4–8 valves, the uncompressed liquid volume of 5 ​nL (2 valves), 10 ​nL (4 valves) and 15 ​nL (6 valves) are forced to move back and forth through the funnel-shaped microfluidic channel (Fig. 1b and Fig. S3). For example, to confine certain liquid volume, valve 1, 4, 5 and 6 have to be firstly closed, i.e., state 011000. To move the liquid volume from left to right, valve 2 and 3 are pressurized, valve 4 and 5 are released, i.e., state 000110. Repeating the cycle leads to reciprocating flow field, driving RBC to repeatedly move through constriction (Fig. S3). Notably, after being pressurized, it takes approximately 50 ​ms for the membrane valve to fully close. The average flow rate can then be estimated by dividing the driven liquid volume, which is associated with the number of valves involved (i.e., 2, 4, and 6), over the timespan of ∼50 ​ms. Using 10 ​μm diameter particles as trackers and 100 fps real-time microscopic imaging, we observe that the peak flow velocity at the center of the constriction is ∼3.42 ​mm/s for 2 valves, ∼15.23 ​mm/s for 4 valves, and ∼41.37 ​mm/s for 6 valves. The number of operating valves also determines the portion of RBCs being forced through the constrictions (Movie. S1and Movie. S2). To ensure uniform mechanical stimulation for all RBCs, the alternative operation of 6 valve is employed for all studies. Our results demonstrate that when the interval between valve operation steps is 50 ​ms, i.e, the time required for one cycle is 200 ​ms, flow rate at the center of constriction can hardly drop down to zero possibly due to inertia (Fig. 1c and d). While, with longer interval (i.e., 500 ​ms), flow velocity return to zero (Fig. 1e). To increase throughput of the blood test, 6 funnel-shaped microfluidic channels are integrated into the microfluidic device (Fig. S1).

### 3.2 Response of RBCs to mechanical stimuli of various constrictions

**Fig. 2:**
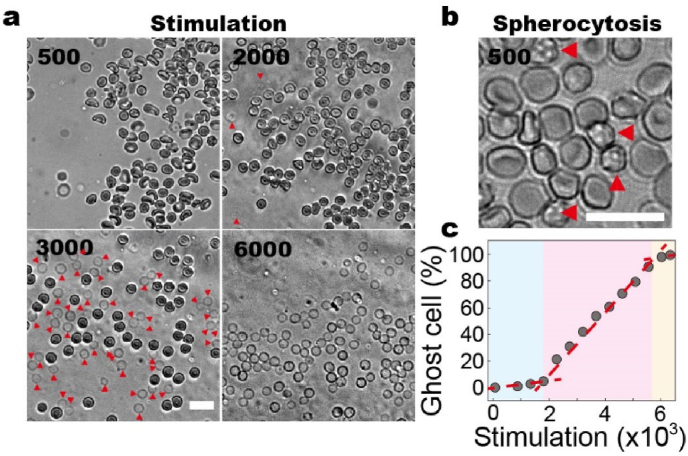
Repeated mechanical stimulation causes hemolysis among RBCs. a. Our results demonstrate that with increased number of mechanical stimulation (i.e., the gradient pressure when repeatedly passing through constrictions), the number of ghost cells increases, which are marked by red triangular markers. b. After 500 times mechanical stimulation, there are a few RBCs showing a more spherical shape, indicating disrupted cytoskeleton structure. c. By counting the number of ghost cells after each mechanical stimulation, our results demonstrate that response of RBCs can be categorized into 3 phases, i.e., the lag phase, the growth phase and the plateau, which are color-coded. All scale bars denote 20 ​μm.

Blood samples from healthy volunteers are collected and diluted by 10,000 times in 0.9 ​% Saline before being loaded into the microfluidic chip (Fig. S4). Real-time monitoring reveals a gradual progression from RBCs expansion to hemolysis (Fig. S5). At lower oscillation frequencies, RBCs initially undergo expansion, which results in the disappearance of their characteristic biconcave disc shape. As the oscillatory shear stress increases, RBCs gradually lose their structural integrity and become increasingly transparent, culminating in the formation of blood shadows. Deformation of RBCs is observed after being forced through 5 ​μm constrictions for ∼500 times, resulting in a more spherical shape (spherocytosis) (Fig. 2b). It has been reported that such morphological changes are due to the disruption of the cytoskeleton. ^32^ As the number of mechanical stimulations increases up to ∼2000, the repeated mechanical stress causes the RBC membrane to rupture, leading to hemolysis (Fig. 2a). This results in the release of hemoglobin and other intracellular contents into the surrounding fluid. The affected cells are essentially devoid of their internal contents, leaving only the RBC membrane, which are often known as “ghost cells.”

**Fig. 3:**
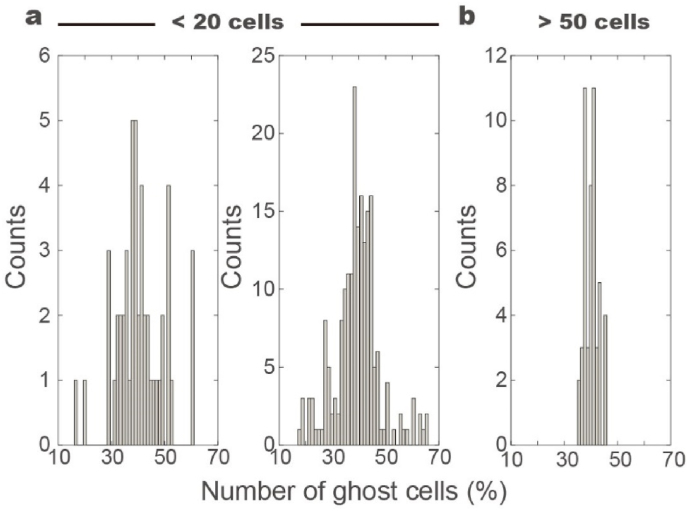
RBC population determines accuracy of the tests. a. The tests, where less than 20 RBCs are forces through 5 ​μm constrictions, are repeated for 50 times. The percentage of ghost cells are counted, showing averaged value and standard deviation of 40±9%. b. The tests containing >20 RBCs are repeated for 200 times, and the values are 39±9%. c. When the number of RBCs is greater than 50 in each group, distribution of the counts for ghost cells is 40±2%.

The increase in the number of ghost cells over time can be categorized into three distinct phases: the lag phase, the growth phase, and the plateau (Fig. 2c). For control samples, the lag phase can be last up to 2000 cycles, during which few RBCs undergo hemolysis. In the growth phase, the number of ghost cells increases linearly with mechanical stimulation, the slope of this increase is shown to correlate with both physiological and pathological conditions. Our results demonstrate that the sampling size significantly impacts the results. When the number of RBCs in one chamber is smaller than 20, the averaged percentage of ghost cells is ∼40 ​% after being forced through 5 ​μm constriction for 3000 times, showing standard deviation of ∼9 ​%, i.e., 40±9% (Fig. 3a). With few cells, increased number of tests up to 200 does not increase the accuracy (Fig. 3b). While, increased number of RBCs in the testing chamber leads to decreased standard deviation value and nearly unchanged percentage of ghost cells, i.e., 40±2%. These findings suggest that, while mechanical stimulation in the microfluidic environment may vary spatially and temporally, repeated stimulation (up to thousands of cycles) ensures that each RBC experiences similar dynamic mechanical conditions. Furthermore, individual RBCs exhibit varying responses to repeated mechanical stress, but statistical analysis shows that results from samples with more than 50 RBCs are highly consistent.

In the plateau phase, the number of ghost cells stabilizes, indicating an equilibrium between the rate of new ghost cell formation and the potential removal or stabilization of these cells. These findings emphasize the critical role of sample size in determining both the rate and extent of RBC deformation and subsequent hemolysis. By pushing RBCs toward hemolysis, we are able to observe early-stage cellular changes that can indicate underlying disease processes. Real-time monitoring of RBC morphology under these dynamic conditions provides valuable insights into the mechanical properties of RBCs and underscores the potential of microfluidic technology in developing diagnostic tools for early detection of diseases, such as diabetes, that affect RBC morphology and mechanics.

### 3.3 Glucose level and age as determining for RBC hemolysis

**Fig. 4:**
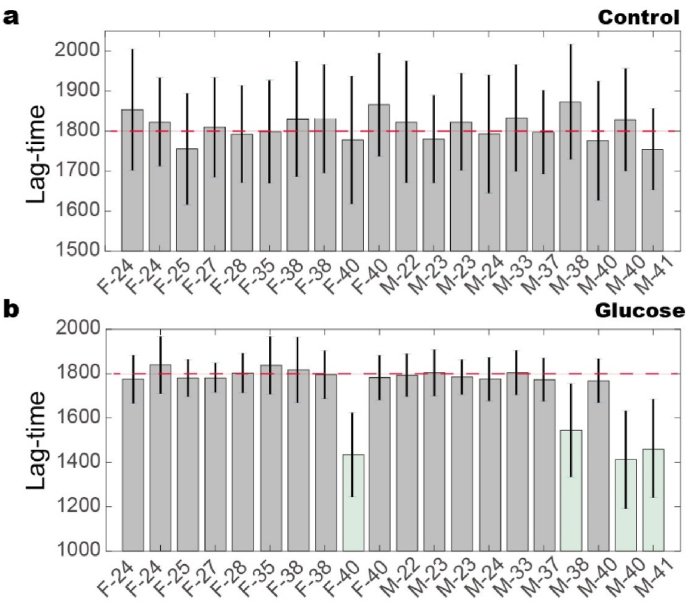
The lag-time of RBCs responding to mechanical stimulation varies over 20 volunteers. a. The lag-phase for different people shows no clear differences. The averaged value is approximately 1800 times of repeated mechanical stimulation. b. After being treated with 20 ​mM glucose for 24 ​h, differences are observed from several blood samples, i.e., one female at the age 40, and 3 males, who are 37, 40 and 41, respectively. The red dashed lines are drawn to compare between the control and glucose-treated samples.

Given the high degree of heterogeneity in RBC responses to mechanical stress under pathological conditions, this study investigates the mechanical properties of RBCs in a simulated pathological environment using a constant glucose concentration (20 ​mM). We collect blood samples from 20 healthy volunteers, consisting of 10 females and 10 males across different age groups. Each blood sample is tested 20 times using a microfluidic chip to obtain average values and standard deviations. The results revealed that the lag-time for RBC hemolysis showed no significant differences among the various groups, with the lag-time of ∼1800 times of mechanical stimulation (Fig. 4a). Treatment with glucose (20 ​mM) for 24 ​h did not produce noticeable differences in most samples (Fig. 4b). Notably, one female at the age of 40 and three males at the age over 38 show a ∼300 times shortening of lag-time. These results indicate that even though the mechanical properties of RBCs remain unchanged at physiological conditions, the response of RBCs to pathological conditions (i.e., the high glucose level) can be distinctive for each person, and age seems to be an important factor. ^33^

**Fig. 5:**
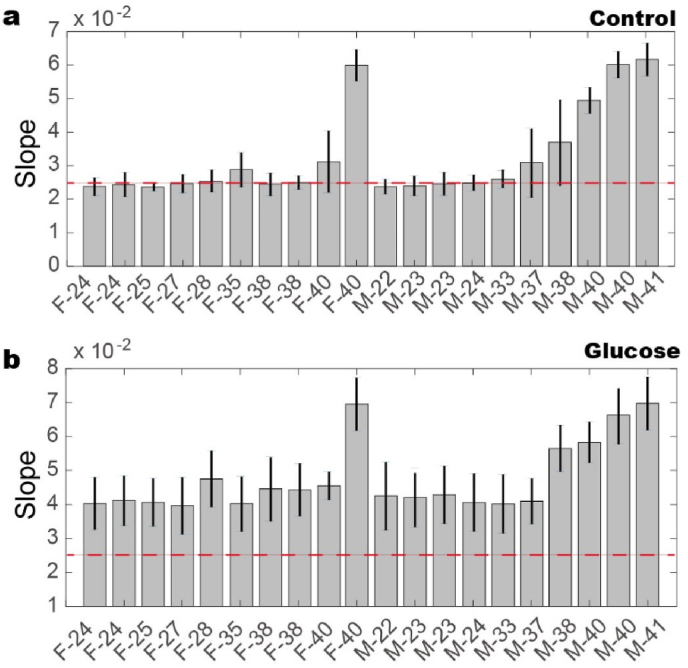
The slope of growth rate for RBCs hemolysis during growth phase. a. Blood samples from aged group show higher values as compared to the young one, i.e., one female and five males. b. After being treated with 20 ​mM glucose for 24 ​h, even though all blood samples show sharper slope of ∼60 ​% increase as compared to the control samples, the differences among groups remain mostly unchanged. The red dashed lines are drawn to compare between the control and glucose-treated samples.

The differences among groups, particularly in terms of sex and age, become more pronounced when the growth slopes of hemolysis rates are compared (Fig. 5). Notably, the growth slope for older individuals is significantly steeper than that for younger ones, i.e., ∼2.5 as compared to ∼5. Additionally, only one female, compared to five males, exhibits an elevated growth slope (Fig. 5a). When blood samples are treated with high levels of glucose, the growth rate increases by ∼60 ​% across all samples (Fig. 5b), and the slope becomes even steeper. Interestingly, glucose treatment does not create discernible differences between the groups—those with initially higher growth rates continue to show higher rates after treatment. These results indicate that age significantly affects the mechanical resilience of RBCs, with older individuals showing a faster onset of hemolysis under mechanical stress. The accelerated growth rate in older groups may be attributed to the cumulative effects of aging on the cytoskeletal integrity of RBCs. However, no such distinction is observed, when the results are categorized according to sex.

Intriguingly, the differences for the lag time among different groups is far less significant as compared to the growth slopes of hemolysis rates. We suspect that during the initial phase of mechanical stimulation (i.e., <1000 times), mechanical stress primarily disrupts the cytoskeleton structure, causing spherocytosis. The cytoskeleton disruption likely leads to an initial adaptation phase where cells alter their shape to a more spherical form, but this process does not immediately result in significant cell rupture. As the number of mechanical stimulations increases, the weakened cytoskeleton structure becomes less capable of maintaining cellular integrity, leading to an accelerated rate of hemolysis, reflected in the steep growth slopes observed. Consequently, the lag time remains relatively constant, as the cells undergo initial morphological changes without immediate rupture. In contrast, the growth rate of hemolysis rates is more sensitive to the cumulative damage from sustained mechanical stress, underscoring the differential cellular responses during the lag and growth phases.

## 4 Conclusion

This study introduces a microfluidic device designed to assess the mechanical properties of RBCs. The device generates a controlled shear flow field, enabling the measurement of morphological changes in RBCs. Using this device, we investigated the mechanical properties of RBCs in different populations and identified a correlation between RBC deformation and both age and blood glucose levels. In subsequent research, we plan to include RBCs from diabetic patients and directly compare them with healthy RBCs, with the goal of optimizing our platform for early diabetes screening.

Compared to conventional diagnostic techniques, such as FPG and HbA1c tests, the mechanical properties of RBCs offer an additional diagnostic parameter that is sensitive to early-stage diabetes, which may not be detectable by conventional biochemical markers.

The microfluidic device presents several advantages: it requires only a small volume of diluted blood, and provides rapid results without the need for extensive sample preparation, and supports high-throughput analysis. These features make it an attractive solution for large-scale clinical screening. This system thus offers a promising, high-throughput diagnostic solution for widespread clinical use.

## CRediT authorship contribution statement

**Yibo Feng:** Writing – original draft, Methodology, Investigation. **Bingchen Che:** Software, Methodology, Investigation. **Yonggang Liu:** Visualization, Methodology, Investigation. **Cangmin Zhang:** Methodology, Investigation. **Jiameng Niu:** Methodology, Investigation. **Jiangcun Yang:** Writing – original draft, Data curation. **Guangyin Jing:** Methodology, Conceptualization. **Dan Sun:** Writing – original draft, Data curation. **Xiaobo Gong:** Writing – original draft, Data curation. **Ce Zhang:** Writing – review & editing, Writing – original draft, Supervision.

## Ethical approval

Accordance with the Declaration of Helsinki, informed consent was obtained from the donors, and the study was approved by the Ethics Committee of Shaanxi Provincial People's Hospital [(2020) R005].

## Declaration of competing interest

The authors declare that there is no conflict of interest regarding the publication of this paper.
