# Supplementary material for: Early diabetes screening via red blood cell mechanics using microfluidic chip integration
Source: Mechanobiol Med. 2025 May 29;3(3):100136. doi: 10.1016/j.mbm.2025.100136 (PMC12179702; doi:10.1016/j.mbm.2025.100136)
Supplement: Multimedia component 1 [file mmc1.docx]

Supporting Information

**Early Diabetes Screening via Red Blood Cell Mechanics Using Microfluidic Chip Integration**

Yibo Feng*^,1^, Bingchen Che*^,1^, Yonggang Liu*^,2^, Cangmin Zhang^3^, Jiameng Niu^4^, Jiangcun Yang^4^, Guangyin Jing^5^, Dan Sun^1^, Xiaobo Gong^&,6^, Ce Zhang^&,1^

^1^*State Key Laboratory of Photon-Technology in Western China Energy, Institute of Photonics and Photon-Technology, Northwest University, No. 1, Xuefu Avenue, Xi’an, 710127, Shaanxi, China*

^2^*Laboratory of Stem Cell and Tissue Engineering, Chongqing Medical University, 400016, Chongqing, China*

^3^*Suzhou Wanguiyuan Precision Technology Co., Ltd., No. 169 Liaobang Road, Suzhou, 215217, Jiangsu, China*

^4^*Department of Transfusion Medicine, Shaanxi Provincial People’s Hospital, Xi’an 710068, Shaanxi, China*

^5^*School of physics, Northwest University, No. 1 Xuefu Avenue, Xi’an, 710127, Shaanxi, China*

^6^*School of Ocean and Civil Engineering, Shanghai Jiao Tong University, Shanghai, 200240, China*


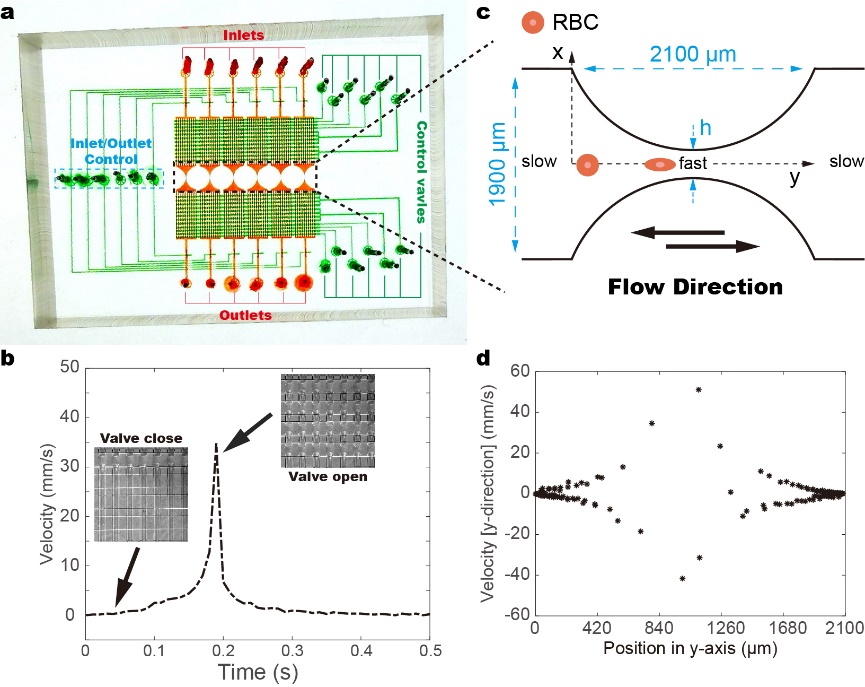


Figure S1: The membrane valve driven fluid generates strong shear forces in a hyperbolic symmetrical microchannel. a. The hyperbolic symmetrical chip filled via the food color (red is the flow layer, and green is the control layer). b. Variation of the velocity of particles in the microchannel when the valve membrane is open and closed (inset shows the valve membrane close and open around the peak, respectively). c. Hyperbolic symmetrical microchannels are 1900 μm wide and 2100 μm long, with a variety of sizes at the narrowest h (e.g., 5, 10, 15, 20, ..., 100 μm). The Flow direction is changed via two side membrane valves of the microchannel. d. The flow velocity of the channel along y-axis and the positive velocity is specified as along the y-axis, whereas the opposite is negative.


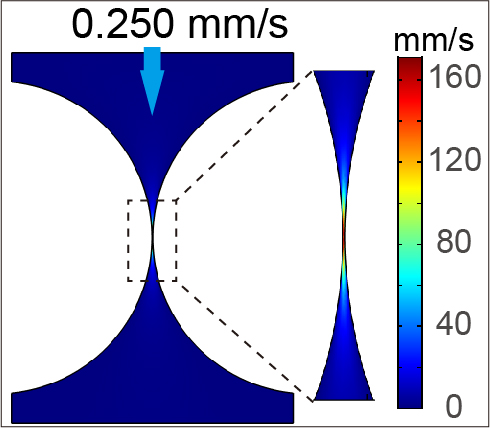


Figure S2: Numerical simulation shows that flow velocity reaches maxima at the center of the funnel-shaped microchannel.


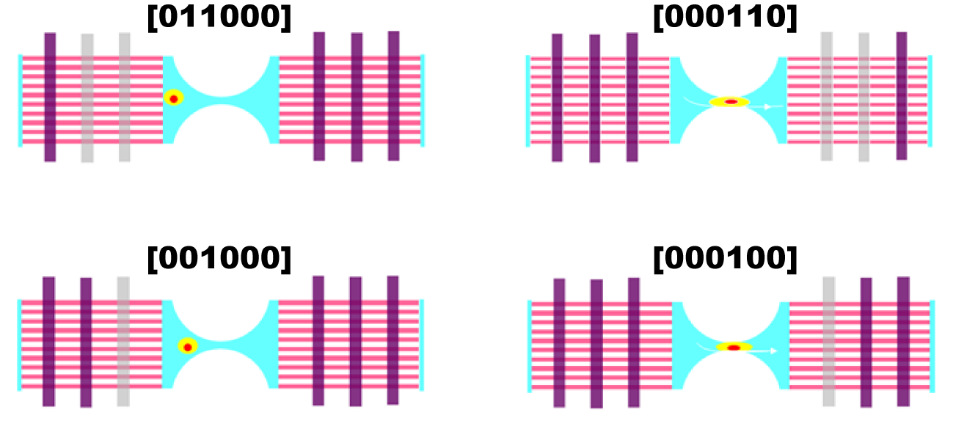


Figure S3: Schematic demonstrates that amplitude of the reciprocating flow can be controlled by changing the number of involved valves. In the top panel, the open/close of 4 valves (i.e., 2 at the right and 2 at the left) drives cell to repeatedly pass through the constriction. While, in the lower panel, only 2 valves are operated.


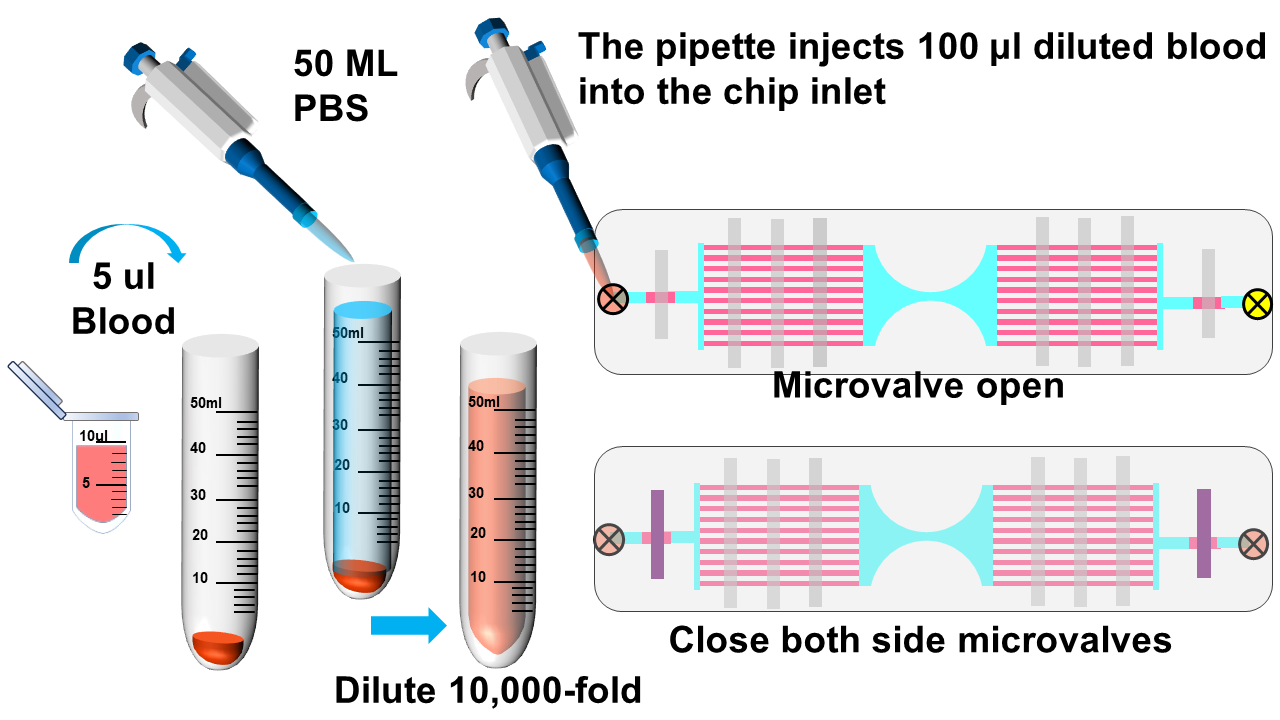


Figure S4: Schematic shows that 5 μl of blood sample is diluted to 50 ml using 0.9% saline, following which 100 μl of the diluted blood sample is loaded into the microfluidic chip via pipetting.


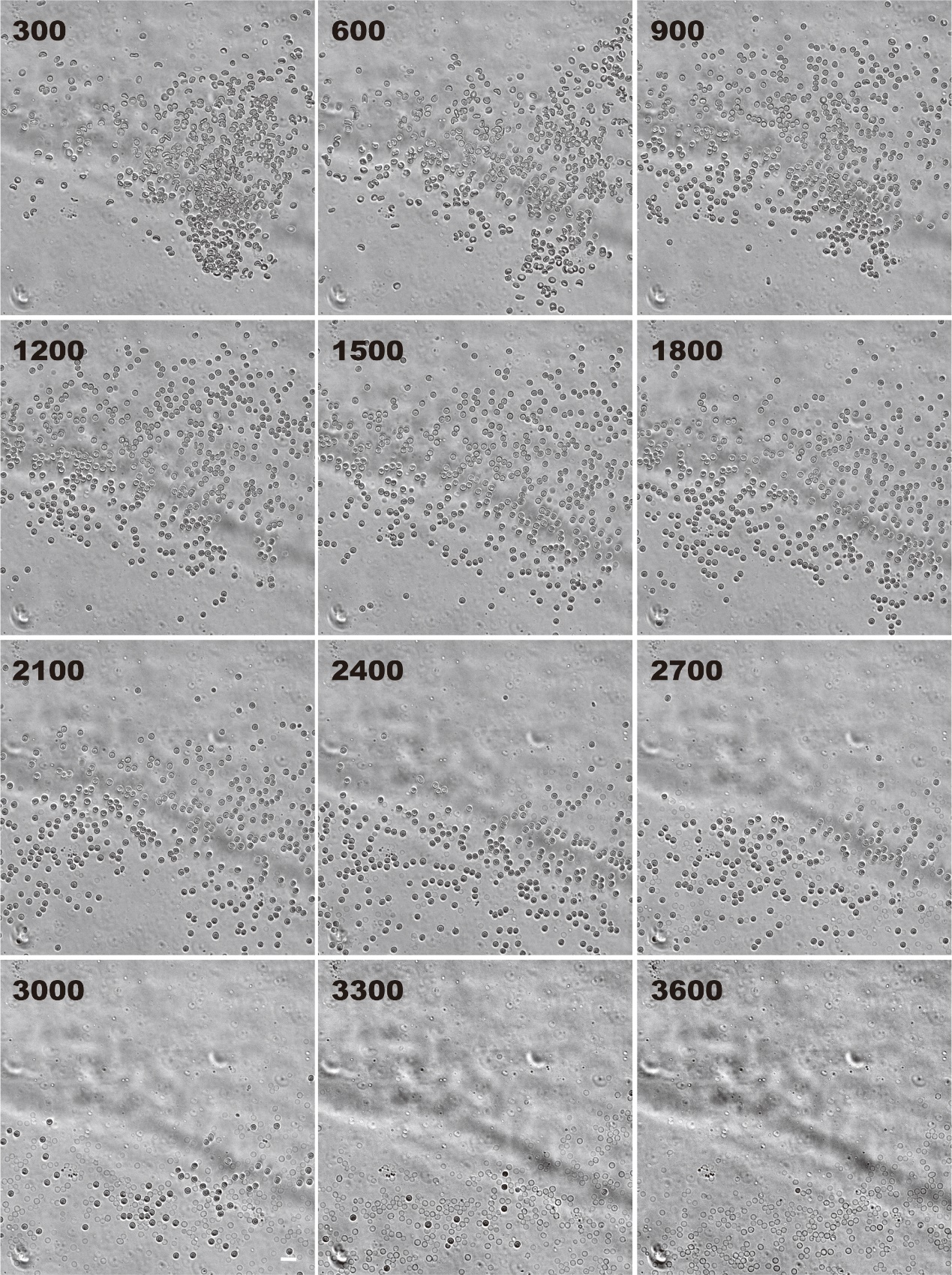


Figure S5: RBCs morphological changes during the frequency increase process. Initially, RBCs maintain their typical biconcave shape, but as the frequency increases, they begin to expand, and the biconcave shape gradually disappears. With further increases in shear frequency, RBCs progressively lose their structural integrity and become increasingly transparent, indicating the onset of hemolysis.

Movie S1: By controlled opening and closing of 2 valves (one on each side), the uncompressed liquid volume of 5 nL are forced to move back and forth through the funnel-shaped microfluidic channel.

Movie S2: By controlled opening and closing of 6 valves (three on each side), the uncompressed liquid volume of 15 nL are forced to move back and forth through the funnel-shaped microfluidic channel.
